# Environmental Pesticide Exposure in the Etiology of Pediatric Brain Tumors and Leukemia: A Scoping Review of Epidemiological Studies

**DOI:** 10.1002/ijc.70546

**Published:** 2026-05-26

**Authors:** Grace N. VanDeSteeg, Alyssa R. Russum, Matthew R. Sandbulte, Eleanor G. Rogan, Martha G. Rhoades

**Affiliations:** ^1^ School of Natural Resources University of Nebraska‐Lincoln Lincoln Nebraska USA; ^2^ Child Health Research Institute University of Nebraska Medical Center Omaha Nebraska USA; ^3^ College of Public Health University of Nebraska Medical Center Omaha Nebraska USA

**Keywords:** genetic susceptibility, household insecticide use, parental pesticide exposure, pediatric cancer, residential proximity to agriculture

## Abstract

Pediatric cancer is a significant cause of morbidity and mortality in children. The etiologies of pediatric cancer are largely unknown, but environmental pesticide exposures are likely to contribute. Chronic low‐dose exposure to pesticide mixtures through drinking water is a growing concern in agricultural communities. This review examines epidemiological studies published between January 1980 and September 2022 that have evaluated the relationship between pesticide exposure and the risk of childhood brain tumors and leukemia. Exposures to pesticides used in the home or ingested through drinking water, residential proximity to agricultural areas where pesticides are used, parental pesticide exposure, and metabolic genotypes are discussed. Findings have shown increased risks for childhood cancers in areas of high agricultural crop density, which may imply a link to increased pesticide applications and pesticide drift from neighboring farm fields. Noted knowledge gaps include the contribution of genetics, exposure through drinking water, individual‐level exposures, and pesticide mixtures to the risk of pediatric cancer. Identifying distinctive genetic traits that influence the metabolism and detoxification of pesticide formulations, and their transformation products is crucial. This knowledge could inform preventive strategies and personalized interventions to reduce the burden of pediatric cancer and protect children's health.

## Graphical abstract

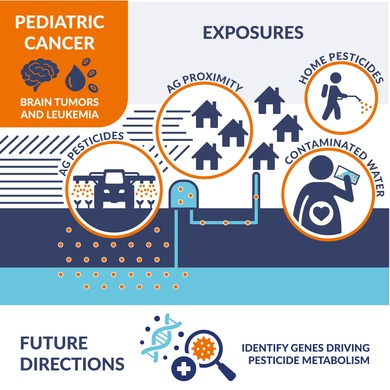

**Abbreviations**
ALL: acute lymphocytic leukemia
AML: acute myeloid leukemia
CBT: childhood brain tumor
CNS: central nervous system
CYP: cytochrome P450
DEET: N‐diethyl‐meta‐toluamide
GST: glutathione S‐transferase
MCL: maximum contaminant level
NAT2: N‐acetyltransferase 2
NQO1: NAD(P)H quinone oxidoreductase 1
PON1: protein paraoxonase 1
PWS: public water system
RR: rate ratio
SDWA: Safe Drinking Water Act
SNP: single‐nucleotide polymorphism
U.S. EPA: United States Environmental Protection Agency

## 1 Introduction

Approximately 15,780 children (ages 1–19 years) are diagnosed with cancer each year in the U.S. [1]. Pediatric cancer is the second leading cause of death in children 5–9 years of age and the third leading cause of death in children ages 10–14 [2]. The national incidence rate for all types of pediatric cancer diagnosed under the age of 20 is 18.8 per 100,000, ranging from 15.3/100,000 in Mississippi to 22.7/100,000 in Maine [3]. Incidence rates are increasing in several agricultural states, including Nebraska, Iowa, Minnesota, Kansas, Illinois, Ohio, and Missouri [3]. The total cost incurred for one child with cancer can approach a million dollars when accounting for medical expenses and lost parental income [4].

Leukemias are cancers originating in the blood‐forming tissues, such as the bone marrow and the lymphatic system. In leukemia patients, the bone marrow produces excessive white blood cells that do not function properly [5]. Leukemia is the most common type of cancer diagnosed in children ages 0–14, and 25%–35% of all childhood cancers are acute lymphocytic leukemia (ALL) [6, 7]. Approximately 5% of cases are believed to have genetic etiologies, but most are thought to result from genetic damage induced by environmental exposures [4]. While the five‐year survival rate has increased with improved treatment, the diagnosis of pediatric leukemia has increased by about 35% over the past four decades [4].

Brain cancer is not only the second most common cancer diagnosed in children, but the number one disease‐related cause of child mortality in the U.S. [6, 8]. Childhood brain tumors (CBTs) are masses of abnormal cells in the brain or tissues and structures surrounding the brain. Random gene mutations are thought to cause most CBTs, but little is known about why the mutations occur or how they lead to tumor formation.

Given the short latency period (time between exposure and symptoms of disease) inherent in pediatric cancer, carcinogenesis may be uniquely linked to high‐intensity environmental exposure and/or highly susceptible individual genotypes [9]. There may also be an epigenetic component where parental exposure increases genetic susceptibility to cancer in children [10]. Residential or agricultural pesticide use in spaces where children spend most of their time poses a significant risk to their well‐being and physical development [11, 12, 13]. Different types of pediatric cancers have varying latency periods, with bone cancers tending to occur in older children. In contrast, blood and brain cancers typically occur in children below the age of 10 [14]. Heredity is expected to play a role, but geographic disparities suggest that pediatric cancer may be affected by something other than inherited genetics, and something is disproportionately affecting the young population [3].

Children's bodies respond to environmental exposures differently from those of adults, partly because their capacity to detoxify chemicals is more limited, and the susceptibility of target organs changes during development [15]. Aside from the vulnerability of the developing fetus to the placental transfer of low molecular weight and lipophilic compounds, a mother's exposure before conception can have a profound effect on her offspring [15]. Identifying links between children and their environments can potentially help prevent childhood cancer in the future. This scoping review was conducted to identify and evaluate the literature related to risk for leukemia and CBTs associated with exposure to pesticides and make recommendations for future research. Our objective was to answer the following research questions:

1. What are the known associations between children's risk of leukemia and brain tumors and exposure of them or their parents to pesticides, transformation products and pesticide mixtures?
2. What are the known associations between children's risk of leukemia and brain tumors and their exposure to pesticides through drinking water?
3. What is known about the contribution of genetics to children's risk of developing leukemia or brain tumors after exposure to pesticides?

## 2 Scoping Review

### 2.1 Search Strategy, Eligibility and Inclusion Criteria, and Selection of Sources of Evidence

A scoping review was conducted using the Preferred Reporting Items for Systematic Reviews and Meta‐Analysis extension for Scoping Reviews (PRISMA‐ScR) [16].

The PubMed and Academic Search Premier databases were utilized between August 2021 and September 2022 to identify relevant peer‐reviewed literature for this scoping review. The terms pediatric brain cancer, pediatric cancer, pesticides, environmental risks for pediatric cancer, acute lymphocytic leukemia, risk factors, gene–environment interaction, metabolism of pesticides, agrochemicals, and water were used in varying combinations. References cited in the publications were scanned for related articles.

Inclusion criteria for the search of publications included as follows: (1) literature reviews including the search terms, (2) meta‐analyses, and (3) epidemiological studies on leukemia and brain cancers. Central nervous system tumors were included if the study referred to brain tumor or inferred brain tumor in the text. This resulted in a total of 5104 potential references. Specific chemical types were also used, including nitrosamines, atrazine, nitrate, and nitrite, which were then added to ensure relevance.

**FIGURE 1:**
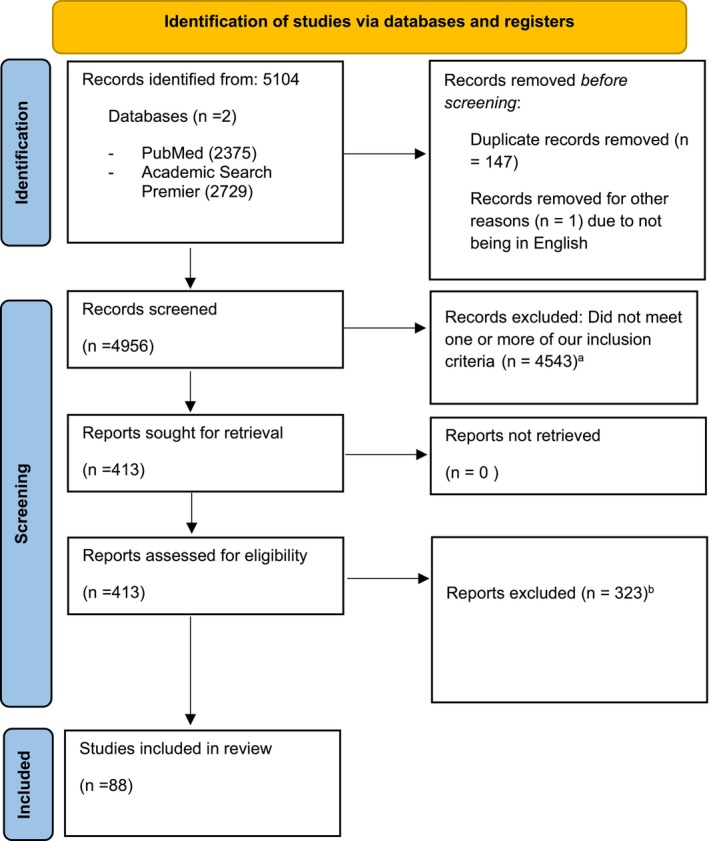
PRISMA flowchart illustrating the selection process of the included studies. (a) 4543 reports were excluded due to: Proxy exposure without specification of exposure, full text unavailable, insufficient data, outside the predefined publication window, linkage between residence and exposure window is missing, pesticide exposure missing, effect estimates not reported or calculable, children and adults combined or children not studied, animal/in vitro studies, agriculture/pesticide exposure not included, not specific to pediatric leukemia/brain tumor, inadequate information (b) 323 reports were excluded due to lack of relevance to pediatric brain tumors or leukemia.

Any papers not including pesticides, not addressing the health of humans, or not published in English were excluded. Further exclusion criteria included the absence of mentions of brain‐specific cancers and leukemia. Papers published before 1980 often grouped heterogenous tumor types which tends to increase misclassification bias. Many pesticide formulations used before 1980 were banned or seldom used after the onset of regulation in the 1970s. It should also be noted that the Food and Drug Administration did not enact the Food Quality Protection Act until 1996, which instituted that the U.S. EPA establish pesticide residue tolerances with a “reasonable certainty of no harm” for infants and children, increasing the safety factor. All documents contained some reported data (Figure 1).

Eighty‐eight papers met the criteria for research on pediatric cancer and environmental pesticide exposure. Common themes of exposure within the home or environment, parental occupation, and potential genes involved were all found to have links to pediatric leukemia and/or CBT. Exposures include direct exposure of the child and parental exposure. Here we discuss the 30 studies reporting statistically significant associations and make recommendations for future research.

### 2.2 Residential Exposures

The home environment can be the source of various chemical exposures during fetal development, infancy, and childhood (Table 1). Parents with occupations in the agricultural industry can bring home trace amounts of chemicals on their shoes and clothing, which can lead to child exposure through inhalation, dermal contact, or ingestion. Children are also exposed through contact with pets treated with insecticides to prevent/kill fleas and ticks [20]. *N, N‐*diethyl‐meta‐toluamide, better known as DEET is the active ingredient in some traditional insect repellents applied to the skin. In rural areas, playing outdoors can expose children to more agricultural pesticides than children living in urban settings might experience. Researchers also propose that maternal ingestion of agricultural chemicals can affect fetal development and increase the risk of brain tumors. This is due to the ability of pesticides to cross the placental barrier [21].

**TABLE 1:** Studies associating pre‐ and post‐natal in‐home exposure to residential insecticides and risk for pediatric brain tumors or leukemia.

Author, year/geographic location/study design/diagnosis year(s)	Number of study subjects	Cancer Type	Exposed person/period of exposure	Odds ratio (95% CI)	Exposure	Exposure pathway
Chen et al. [11]/international/Meta‐analysis/1985–2011	N/A	Total Leukemia	Males and females ≤ 20 years of age	1.47 (1.26–1.72)	Insecticides	In‐home application
				1.26 (1.10–1.44)	Herbicides	Outdoor application
Greenop et al. [17]/Australia/case–control/2005–2010	Cs/ctrl 72/187	CBT*	Mother/father	1.54 (1.07–2.22)	Any pest control treatment in the home or garden	Professional extermination
	17/31		Year before pregnancy	2.17 (1.12–4.19)	Termite treatment	
Ma et al. [18]/California/case–control/1995–1999	152/129	Leukemia	Year 1 of life	2.3 (1.1–4.9)	Insecticides, flea control products, and herbicides	Professional extermination
	140/120		Year 2	3.6 (1.6–8.3)		
	116/98		3 months before pregnancy to year 3	2.8 (1.4–5.7)		
	140/120	ALL*	Year 2	3.3 (1.4–7.7)		
	116/98		3 months before pregnancy to year 3	2.6 (1.2–5.4)		
	162/135	Leukemia	3 months before conception	1.8 (1.1–3.1)	Insecticides (excluding flea control products)	Personally applied
	162/135		During pregnancy	2.1 (1.3–3.5)		
	116/98		3 months before pregnancy to year 3	2.1 (1.1–4.3)		
	162/135	ALL*	During pregnancy	2.3 (1.3–4.0)		
	116/98	Leukemia	3 months before pregnancy to year 3	2.4 (1.2–5.1)	Insecticides	Applied > 5 times
Pogoda et al. [17]/California/case–control/1984–1991	Cs/Ctrl 76/53	CBT*	Prenatal and childhood	1.7 (1.1–2.6)	Flea/Tick pesticides	
	33/17		Prenatal (subjects age 0–19 years)	2.2 (1.1–4.2)		Product prepared/cleaned up by mother
	10/3		(age 0–4 years)	5.4 (1.3–22.3)		
	17/6		(subjects age 0–19 years)	10.8 (1.3–89.1)		Spray/fogger
	30/17		(subjects age 0–19 years)	2 (1.0–4.0)		Product applied to > 1 pet
	11/5		(age 0–4 years)	3.5 (1.1–11.4)		
	30/17		(subjects age 0–19 years)	1.9 (1.0–3.8)		Product applied every 1–2 months
	12/4		(age 0–4 years)	4.4 (1.2–15.5)		
Infante‐Rivard et al. [19]/Case–control/Quebec, Canada/1980–1993	Cs/Ctrl 491/491	ALL*	Mother (1 month before to end of pregnancy)		Insecticides	Used in the home
	168/111			1.79 (1.34–2.40)	Cockroaches, ants, flies, bees, wasps	
	45/20			2.47 (1.43–4.28)	Moths	
	96/67			1.59 (1.11–2.26)	Insects	
	212/170		Child (0–9 years)	1.38 (1.07–1.77)	Cockroaches, ants, flies, bees, wasps	
	50/25			2.13 (1.30–3.47)	Moths	
	137/87			2.99 (1.42–2.82)	Insects	
	118/71		Mother (1 month before to end of pregnancy)	1.84 (1.32–2.57)	Pesticide class Herbicides	Used in garden, yard and indoor plants
	78/42			1.97 (1.32–2.94)	Plant insecticides	
	63/39			1.70 (1.12–2.59)	Tree products	
	178/144		Child (0–9 years of age)	1.41 (1.06–1.86)	Herbicides	
	128/84			1.82 (1.31–2.52)	Plant insecticides	
	99/76			1.41 (1.01–1.97)	Tree products	

* *CBT‐Childhood brain tumors; ALL‐ Acute Lymphoblastic Leukemia*.

In‐home application of insecticides—a subset of pesticides—leads to exposure to chemicals such as sulfuryl fluoride, methyl bromide, pyrethroids, organophosphates, and carbamates [22]. Such exposures could stem from commercial applications, the use of flea and tick treatments on pets, the application of insecticides to household plants, and the use of insect repellents. A 2015 meta‐analysis reported childhood exposure to insecticides used in the home was associated with increased risk of leukemia [11]. Residential pest control treatments during the 12 months before conception were associated with a 1.5‐fold increase in CBT risk based on data collected through written and telephone questions. The risk was also higher when the pest control treatment was before pregnancy only. The risk for high‐grade glioma was highest, over 4‐fold above baseline, when pest control treatments were performed during pregnancy [17].

An increased risk for pediatric brain tumors was associated with prenatal exposure to flea and tick products, especially for children under 5 years of age at diagnosis [20]. This study, which included 224 cases and 218 controls residing in Los Angeles County, reported a 2.2‐times higher risk for children whose mothers prepared, applied, or cleaned up flea and tick products. Risk increased with the number of pets treated. No elevated risk was observed for pesticides used to treat termites, lice, nuisance pests, yard or garden insecticides, herbicides, fungicides, or snail killers [20]. Specific active ingredients in the pesticide products were not identified.

The critical window of susceptibility—the time during development when the organism is most vulnerable—is essential in pesticide toxicity. Animal studies indicate that prenatal and postnatal pesticide exposures may lead to childhood brain tumors and leukemia through distinct biological mechanisms [13]. The Northern California Childhood Leukemia Study reported that the use of professional pest control services from 1 year before birth to 3 years after was more common among households where a child was eventually diagnosed with leukemia, either in aggregate (*n* = 162) or specifically acute lymphoblastic leukemia (ALL) (*n* = 135) [18]. The use of professional pest control services was associated with a significant increase in the risk of childhood leukemia, with the highest risk (increased threefold) during the second year. In this study of children aged 0–14 years, exposure to insecticides (excluding products for flea control) was more closely associated with leukemia and acute lymphoblastic leukemia (ALL) when the exposure occurred during pregnancy than when it happened in the child's third year [18]. The risk doubled in households that received more than five pesticide applications over a four‐year period, compared to a 1.5‐fold increase in those with one to five applications.

### 2.3 Drinking Water

Agrichemical contamination of drinking water supplies is a growing concern; however, few studies have directly examined pesticides in drinking water as a risk factor for pediatric cancer [23]. Although not a pesticide, nitrate is present in most water supplies and can be considered a surrogate for pesticide contamination. Nitrate‐nitrogen concentrations below 2–3 mg/L are generally considered naturally occurring [24]. Many water supplies are contaminated by nitrate from nitrogen‐based fertilizers applied to farm fields [25]. Fields where nitrogen fertilizer is applied frequently also receive pesticide applications, and soil conditions favorable to nitrate leaching are expected to have co‐occurring pesticide leaching. Table 2 summarizes studies that associate agrichemical exposure through drinking water during pregnancy with the risk for pediatric brain tumors or leukemia.

**TABLE 2:** Studies assessing the association between agricultural exposures from maternal drinking water during pregnancy and risk for pediatric brain tumors or leukemia.

Author, year/geographic location/study design/diagnosis year(s)	Number of study subjects	Cancer Type	Exposed person/period of exposure	Odds ratio (95% CI)	Exposure	Exposure pathway
Zumel‐Marne et al. [26]/Canada, Greece, Italy, New Zealand, Spain, Korea/Case–control/2010–2016	Cs/Ctrl 34/94	CBT*	Pre‐ and postnatal (subjects age 10–24 years)	2.2 (1.02–4.40)	Nitrate ion (${\mathrm{NO}}_{3}^{-}$) concentration 41.7–97.0 mg/L lifetime dose	Tap water (public water system) (mother/child)
Thorpe & Shirmohammadi [27]/Maryland, USA/Ecological/1992–1998	1,218,790	Leukemia	Children age 0–17 years	1.35 (1.02–1.78)	Nitrate, atrazine, metolachlor, simazine, alachor	Groundwater/public water supply wells (maternal/child)
	(293 cases)			1.81 (1.35–2.42)	Nitrate	
Mueller et al. [28]/USA, France, Italy, Spain, Isreal, Canada, Australia/Case–control/1976–1994	Cs/Ctr6/8	CBT*		5.2 (1.2–23.3)	Nitrite ion (${\mathrm{NO}}_{2}^{-}$) ≥ 5 mg/L	Public water, well, or spring (maternal)
	N/R	Astroglial		4.3 (1.4–12.6)	1–< 5 mg/L	
	N/R			5.7 (1.2–27.0)	≥ 5 mg/L	

*Note:* N/*R* = not reported.

* *CBT‐Childhood brain tumors*.

In a 2004 multi‐center study of 185 cases and 341 controls, with nitrate/nitrite water analyses performed by dipstick at the tap, neither nitrate nor nitrite were associated with an increased risk for CBT [28]. Nitrate concentrations of 10–< 20 mg/L nitrate ion (NO_3_) were associated with a decreased risk of approximately one‐half. The association between CBT risk and detectable nitrite at 1– < 5 mg/L was insignificant. Nitrite concentration ≥ 5 mg/L was modest but not significantly associated with increased risk for CBT. When the analyses were restricted to mothers reporting they had not used bottled water during their pregnancies (131 cases and 241 controls), no increased or decreased risk associated with nitrate was observed. Still, nitrite ≥ 5 mg/L was associated with a 5‐fold higher risk for CBT. Nitrite concentrations of 1–< 5 mg/L were associated with astroglia tumors, and risk was increased when nitrite concentrations were ≥ 5 mg/L. No significant association was found between nitrate/nitrite concentrations in drinking water and the overall risk of CBT. However, high nitrite (> 5 mg/L) was linked to increased risk in specific cases, with reliability limited by maternal recall and the dipstick method [28]. The maximum contaminant levels (MCLs) for nitrate and nitrite as ions are 45 mg/L (NO_3_), and 3.28 mg/L (NO_2_), respectively, or 10 mg/L for nitrate‐N (nitrate measured as nitrogen) and 1 mg/L for nitrite‐N (nitrite measured as nitrogen) [29]. Nitrite is unstable in water, and the dipstick detection method is not considered reliable for monitoring concentrations under the United States Environmental Protection Agency's Safe Drinking Water Act (U.S. EPA SDWA). In a 2021 investigation through the MOBI‐Kids case–control study using historical tap water concentrations, there was a two‐fold risk for neuroepithelial brain tumor associated with a lifetime cumulative exposure of 41.7–97 mg/L per day of nitrate, measured as NO_3_ [26].

An ecological study in Maryland explored potential associations between spatial patterns of four types of childhood cancers (leukemia, brain and spinal cord, non‐Hodgkin lymphoma, and bone) and the four most detected pesticides in Maryland groundwater [27]. When children were potentially exposed to a mixture of nitrate, atrazine, and metolachlor, the risk of acquiring one of the four types of cancer was 7.56 times greater than potentially unexposed children and 5.31 times more significant when the mixture contained simazine in addition to nitrate, metolachlor, and alachlor (*p* < 0.0001). The risk of leukemia increased when all four compounds were present in the groundwater. The findings highlight the importance of evaluating agrochemicals, both individually and in mixtures, in drinking water as potential risk factors for cancer development in children. Atrazine is a triazine herbicide, classified as a secondary amine, known to react with nitrite (reduced from nitrate) to form *N*‐nitrosoatrazine in an acidic environment such as the human stomach [30, 31]. Many pesticides and their transformation products contain secondary amine/amide moieties in their chemical structure, giving them the potential to form *N*‐nitroso compounds when in the presence of nitrite [32, 33]. Maternal exposure to nitrosatable medications was associated with increased risk for CBT in a 2003 case–control study conducted in California [34].

### 2.4 Residential Proximity to Farm Fields

Most of the literature on pesticide use and its association with childhood brain cancer and leukemia focuses on exposure modeled through agricultural land use and residential proximity to farm fields (Table 3). There was a statistically significant increased risk for some childhood cancers in counties with moderate (20–< 60%) to high (≥ 60%) total cropland when compared to < 20% cropland [39]. In this ecological study, which included 25 U.S. cancer registries and over 1000 counties, there was no observed association between total cancers and the percentage of cropland when comparing the moderate and high‐risk groups to the referent group (< 20% total cropland). However, leukemia risk was elevated in counties with > 60% cropland. Specifically, lymphoid leukemias and acute myeloid leukemia (AML) were associated with the highest cropland counties. Central nervous system tumor risk was also associated with high cropland counties, particularly astrocytoma, with 1.5 times higher odds for astrocytoma and 1.9 times higher odds for primitive neuroectodermal tumor (PNET). The study evaluated cropland as the number of acres planted to the six leading U.S. crops (barley, wheat, corn, oats, cotton, and soybeans) divided by total acres in the county, followed by calculations for each crop category. As some crops require more than one application or more concentrated formulations, the authors queried for different cancer frequencies in counties with distinct crop profiles. The authors observed no association between these cancers and residence in a county with mainly barley, cotton, or wheat crops. AML risk was increased chiefly in soybean production areas, and an increased risk of central nervous system tumors was associated with residing in a county with oat crops [39]. These findings suggest an increased risk for childhood cancer in areas of high agricultural crop density, which may indicate increased pesticide applications and pesticide drift from neighboring farm fields. Crop density and proximity to farmland are frequently used as surrogates for agricultural pesticide exposure based on supporting evidence examining agricultural pesticide levels in house dust [36].

**TABLE 3:** Summary of studies associating residential proximity to farming, pesticide exposure, and risk for pediatric brain tumor or leukemia.

Author, year/geographic location/study design/diagnosis year(s)	Number of study subjects	Cancer type	Exposed person/period of exposure	Odds Ratio (95% CI)	Exposure	Exposure pathway
Rull et al. [35]/California, USA/Case–control/1995–2002	Cs/Ctrl	ALL*	Child		Exposure (pounds applied/mile^2^)	Application within ½ mile of residence
	41/33			1.7 (1.0–3.1)	Lifetime Fumigants (1–549)	
	60/50			1.6 (1.0–2.6)	Pesticides classified as: Possible carcinogens (1–83)	
	67/55			1.6 (1.0–2.5)	Possible/probable carcinogens (1–161)	
	62/56			1.6 (1.0–2.6)	Suspected genotoxins (1–263)	
	60/49			1.6 (1.0–2.7)	Cholinesterase inhibitors (1–79)	
	41/29			1.9 (1.0–3.4)	First year of life Probable carcinogen (1–106)	
	48/37			1.7 (1.0–3.4)	Possible/probable carcinogens (1–161)	
	10/8			3.9 (1.0–15.7)	Lifetime Pesticides classified as: Azoles (2–21)	
	56/46			1.6 (1.0–2.7)	Organophosphates (1–79)	
	31/24			4.1 (1.5–11.1)	Triazines (1–27)	
Booth et al. [36]/Illinois, Indiana, Ohio, Michigan, Missouri, Iowa, USA/Ecological/2004–2008	518 Cases		Child (< 5 years of age)	**RR** (95% CI)		Residential proximity to farming (% county crop density)
		Total leukemia		1.09 (1.03–1.14)	Dry beans	
				1.11 (1.04–1.19)	Sugar beets	
		ALL*		1.10 (1.04–1.16)	Dry beans	
				1.11 (1.02–1.21)	Sugar beets	
		AML*		2.03 (1.25–3.28)	Oats	
Patel et al. [37]/Denmark/Cohort/1996–2014	61 cases/9171 non‐cases	Leukemia	Mother	**HR** (95% CI)	Peas	Crop density (hectares within 500 m of home)
				2.6^a^ (1.02–6.8)	Total crops	24–66
				2.0 (1.02–3.8)		
				2.4^a^ (1.1–5.3)	Winter cereals	1.5–< 8.3
				2.1 (1.09–4.0)		
				2.7^a^ (1.2–6.2)	Grass/clover	> 0–< 1.1
				3.1^a^ (1.2–7.7)		1.1–32
				2.1 (1.1–4.0)		
				2.4^a^ (1.02–5.4)	Crop	> 0–12
				2.0 (1.03–4.0)		
				2.8^a^ (1.1–6.9)	Maize	> 0–20
				2.3 (1.1–4.9)		
Lombardi et al. [38]/California, USA/Case–control/1998–2011	119/123,158	Astrocytoma	Mother (child age 0–5 years)	2.12 (1.13–3.97)	Bromacil	Pesticide applied within 4000 m of maternal residence
				1.64 (1.02–2.66)	Thiophanate‐methyl	
				2.38 (1.44–3.92)	Triforine	
				2.09 (1.03–4.21)	Kresoxim‐methyl	
		Medulloblastoma		1.78 (1.15–2.76)	Chlorothalonil	
				1.6 (1.02–2.53)	Propiconazole	
				1.6 (1.06–2.43)	Dimethoate	
				2.52 (1.25–5.11)	Linuron	
		Ependymoma		1.72 (1.10–2.68)	Thiophanate‐methyl	
Carozza et al. [39]/USA/Ecological/1995–2001	Cases		Children < 15 years of age			Residential proximity to farming
	1357	Total Leukemia		1.2 (1.1–1.3)		> 60% cropland in county
	1074	Lymphoid leukemias		1.3 (1.1–1.4)		
	207	AML*		1.8 (1.4–2.3)		
	975	CNS tumors		1.3 (1.1–1.4)		
	475	Astrocytoma		1.5 (1.3–1.7)		
	222	PNET*		1.9 (1.5–2.4)		
		AML*		1.4 (1.1–1.7)	**Crop** Soybean	≥ 20% cropland in county
		CNS*		1.1 (1.0–1.3)	Oats	
		Astrocytoma		1.1 (1.0–1.3)	Corn	
				1.3 (1.0–1.6)	Oats	
		PNET*		1.5 (1.1–2.0)	Oats	
				1.2 (1.0–1.5)	Soybean	
Gomez‐Barroso et al. [36]/Spain/Case–control/1996–2011	Cs/Ctrl		Age 0–14 years		Exposed to crops	Residential proximity to farming: percentage of crop surface within 1 km around child's residence (Crop Global Index in quartiles)**
	1062/6451	Leukemia		2.66 (1.98–3.56)	Total Crop	Q2 (2.55%–8.91%)
				2.83 (2.08–3.85)		Q3 (8.91%–26.42&)
				2.53 (1.81–3.51)		Q4 (26.42%–100%)
				1.70 (1.13–2.58)	Irrigated	Q1 (0%–2.55%)
				3.10 (2.19–4.42)		Q2 (2.55%–8.91%)
				1.90 (1.24–2.94)		Q3 (8.91%–26.42&)
				2.13 (1.39–3.30)		Q4 (26.42%–100%)
				1.89 (1.21–2.95)	Heterogenous	Q2 (2.55%–8.91%)
				2.94 (1.98–4.37)		Q3 (8.91%–26.42%)
	711/4255			1.94 (1.23–3.06)		Q4 (26.42%–100%)
				2.96 (1.33–6.57)	Vineyards	Q3 (8.91%–26.42%)
		CNS		2.05 (1.44–2.92)	Total Crop	Q2 (2.55%–8.91%)
				3.36 (2.46–4.59)		Q3 (8.91%–26.42&)
				3.65 (2.66–5.01)		Q4 (26.42%–100%)
				2.29 (1.48–3.57)	Irrigated	Q2 (2.55%–8.91%)
				2.19 (1.40–3.45)		Q3 (8.91%–26.42%)
				3.70 (2.53–5.46)		Q4 (26.42%–100%)
				1.91 (1.17–3.11)	Heterogenous	Q2 (2.55%–8.91%)
				2.79 (1.79–4.36)		Q3 (8.91%–26.42%)
				1.86 (1.11–3.10)		Q4 (26.42%–100%)
				2.55 (1.36–4.80)	Fruits	Q3 (8.91%–26.42%)
				2.94 (1.59–5.43)		Q4 (26.42%–100%)
Shim et al. [40]/Florida, New Jersey, New York and Pennsylvania, USA/Case–control/1993–1997		CBT*	< 10 years of age		Herbicides, insecticides and fungicides Herbicides	
		Astrocytoma				
	53/27			1.9 (1.2–3.0)		Parent used for gardens and lawns
	40/20			2.0 (1.2–3.4)		Father applied to gardens and lawns
	52/26			1.9 (1.2–3.1)		Home use and/or exposure through father's job
	41/22			1.8 (1.1–3.1)		Father applied at home and/or exposed at work
Hyland et al. [41]/Costa Rica/Case–control/1995–2000	Exposed Cs/Ctrl	ALL*	Mother		Insecticides, herbicides and fungicides	Pesticide applications on farms or companies near home
		Boys				
	91/159		Year before pregnancy	1.63 (1.05–2.53)	Insecticides	
	92/157		during pregnancy	1.75 (1.13–2.73)		
	96/163		breastfeeding	1.75 (1.12–1.73)	Spraying	Maternal insecticide use in home
	40/61		during pregnancy	1.67 (1.03–2.71)		
	39/60			1.64 (1.01–2.67)		
	61/92		breastfeeding any time period	1.74 (1.13–2.69)		
		Boys and Girls	any time period	1.52 (1.11–2.09)		
Park et al. [42]/California, USA/Case–control/1998–2011	Exposed cs/ctrl	ALL*	< 6 years of age			Maternal exposure during pregnancy (within 4000 m of residence)
	52/3204			1.62 (1.02–2.56)	**Anilide**	
	13/405			2.21 (1.16–4.22)	Propanil	
					**Amide**	
	22/1177			2.12 (1.06–4.23)	Propyzamide	
					**Organophosphate**	
	76/3757		2.10	2.10 (1.30–3.39)	Phoshmet	
					**Urea**	
	98/5364		1.79	1.79 (1.02–3.15)	Diuron	
					**2,6‐Dinitroaniline**	
		AML*		2.23 (1.38–3.60)	Trifluralin	

*Note:* RR = rate ratio; HR = hazard ratio.

* *CBT‐Childhood Brain tumors; ALL‐ Acute Lymphoblastic Leukemia; AML‐ Acute Myeloid Leukemia; PNET‐ Primitive Neuro‐Ectodermal tumor; CNS‐ Central Nervous System*.

** *The quartiles represent the range of crop exposure as a percentage of land area within 1 km of the home*.

^a^ Adjusted for animals.

A study of 3350 cases and 20,365 controls, aged 0–14, in two regions of Spain reported an increased risk for childhood cancers when living close to agricultural fields (within 1 km) [43]. The total percentage of crop surface (irrigated land, rice fields, vineyards, fruit trees, berry plantations, olive groves, and heterogeneous agricultural areas) within the 1 km buffer was calculated and subdivided into quartiles (Q) for a total crop index and then for each of the six categories. Overall, the risk for developing leukemia from 1.76 times greater for Q1 of total crop surface to 2.83 times greater for Q3. The risk of leukemia in the highest quartile (Q4) of total crop surface was similar at 2.53. Total CNS tumor risk was increased for Q 2–4. Irrigated crop surface was associated with an increased risk for leukemia. CNS tumors were associated with an increased risk for Qs 2–4. Heterogeneous crop surface was associated with an increased risk of leukemia and CNS tumors for Qs 2–4. CNS tumor risk was associated with fruit crops. A vineyard crop surface was associated with a three‐fold increased risk of leukemia in Q3.

An ecological study using county‐level agricultural and cancer registry data linked dry bean farming to total leukemia risk in the Midwest region of the United States [36]. Crop densities for counties in Illinois, Indiana, Ohio, Michigan, Missouri, and Iowa were calculated for barley, dry beans, corn, hay, oats, sorghum, soybeans, sugar beets, and wheat and combined with cancer incidence data to evaluate relationships between crop density and cancer incidence using rate ratios (RR) [36]. Sugar beet crop density was also associated with a slight increase in risk for total leukemia. No significant association was observed for leukemia and soybean crop density, barley, hay, oats, sorghum, or wheat. A positive association was also observed between oat crop density and AML. There was no statistical association for CNS tumors, perhaps due to low cancer incidence in some counties [36]. A study of 213 ALL cases and 268 matched controls enrolled in the Northern California Childhood Leukemia Study reported an increased risk of ALL for children living within ½ mile of pesticide applications [35]. ALL risk was increased when children were exposed to fumigants (OR 1.5; CI 1.0–3.1). Pesticides classified as possible carcinogens were also associated with ALL risk as were pesticides classified as suspected genotoxins and cholinesterase inhibitors when children were exposed during the first year of life. When evaluating pesticides by physicochemical class, there was an increased risk associated with exposure to urea pesticides. Lifetime triazine pesticide exposure was associated with a four‐fold increased risk of ALL (OR 4.1; CI 1.5–11.1) [35].

Lombardi, et al. utilized data from the California Cancer Registry to identify CNS tumor cases [38]. Prenatal pesticide exposure was determined using California's Pesticide Use Reporting system, identifying applications within 4000 m of the mother's residence at the time of the child's birth. The risk for astrocytoma was increased for mothers exposed to the herbicide bromacil and the fungicides thiophanate, triforine, and kresoxim methyl. Medulloblastoma risk was elevated when mothers were exposed to chlorothalonil or propiconazole (both fungicides). The insecticide dimethoate and linuron (a herbicide) showed increased risk. Thiophanate‐methyl was also associated with an elevated risk for ependymoma. In this study of children ages 0–5 years, 66.9% of medulloblastoma cases were male. In all subtypes, mothers were more likely to be Hispanic or White non‐Hispanic than any other race [38].

Shim et al. [40] found a link between brain tumors, especially astrocytoma, residential herbicides used for lawn or garden, and insecticide usage. Herbicide usage showed a two‐fold risk increase when the father applied herbicides. When occupational and residential exposures were combined, the risk of astrocytoma remained elevated, regardless of which parent used the chemicals. Fathers who washed or changed clothes after usage reduced the risk of brain tumors in their children as opposed to fathers who took no precautions after usage [40]. Although imprecise, the results aligned with the residential use of pesticides. There was no association between PNET risk and parental pesticide exposure; however, when fathers applied insecticides in a residential setting or were exposed through their jobs, the risk for all types of brain cancer increased nearly three‐fold [40].

A study in California examined instances of cancer in children under six, focusing on their exposure to pesticides during early childhood or their mothers' exposure during pregnancy. The assessment utilized data from the California Pesticide‐Use Reports and the California Public Land Survey System to calculate monthly and annual application rates within a 4000 m radius of the maternal residence, estimating exposure. Elevated risk was observed for ALL when mothers were exposed to any of the 59 carcinogenic pesticides from the original list of 133 pre‐selected pesticides. Elevated risk was also observed with pesticide exposure in the classes of 2,6‐dinitroanilines, anilides, and ureas. Exposure to pesticides classified as ureas was also associated with increased odds of AML. Diuron, phosmet, kresoxim‐methyl, propanil, glyphosate, and paraquat dichloride were also associated with increased odds for ALL risk when modeled as individual compounds [42].

Children in Denmark were at 2 times increased risk of leukemia when their mothers lived in areas with > 24 ha of total crop area within 500 m of their home [37]. The risk increased to almost 3 times after adjustment for livestock farms within 1000 m of their residence. When the data were modeled to compare mothers living near animal farms, no association with leukemia risk was observed for any livestock animals or for cattle or pigs individually. However, the risk of CNS tumors was elevated when mothers lived within 500 m of cattle farms, although no statistically significant association was observed between animals or crops within 500 m of the home and CNS tumors in children [37].

In Costa Rica, an increased risk of acute lymphoblastic leukemia (ALL) was observed in boys whose mothers reported exposure to insecticides inside the home during the year before pregnancy, during pregnancy, or while breastfeeding [41]. Boys were at increased risk of ALL when mothers reported pesticides being applied near the home during pregnancy or breastfeeding [41]. This study of 251 cases and 577 controls also concluded that mothers with less than a 12th‐grade education were more likely to be exposed than those who completed high school [41]. This could be because those with higher education tend to have a higher socioeconomic status.

### 2.5 Parental Occupational Exposure

Pediatric cancer risks have been associated with parental occupational exposure to pesticides (Table 4). Efird and colleagues [51] examined the farm or agriculture‐related exposures of the children themselves and of mothers. This study of 1218 cases and 2223 controls found an increased risk for pediatric brain tumors in children whose mothers were farmers or farm workers. Specifically, children living on a farm and exposed to horses, cats, and pigs combined had a three‐fold risk of CBT compared to any other animal/combination. Children living on a farm who were exposed to pigs, horses, dogs, or cats were at significantly increased risk of CBT in aggregate. Similar results were observed for children of mothers exposed to these animals. Children's farm exposure to dogs and cats was also associated with an increased risk for PNET. The risk for PNET increased 4 times with maternal farm exposure to pigs. The risk was also elevated for children whose mothers were general farm workers. Compared with no exposure, maternal exposure to nitrogen‐based fertilizers and pesticides was associated with an increased risk of PNET [51]. Exposure to farm animals may serve as a surrogate for pesticide exposure through application of insecticides to the animals or the buildings where animals are housed, or the mixing of pesticides that are used on farm animals or farm fields. It would also be expected that producers working with animals on the farm would be exposed to pesticides used in crop production.

**TABLE 4:** Studies associating pediatric cancer risk with parental exposure to workplace pesticides.

Author, year/geographic location/study design/diagnosis year(s)	Number of study subjects	Cancer type	Exposed person/period of exposure	Odds Ratio (95% CI)	Exposure	Exposure pathway
Feychting et al. [44]/Sweden/Cohort/1976, 1977, 1981 and 1982	235,635	Nervous system tumor	Father/preconception			
	No. cases			RR**		
	11			2.36 (1.27–4.39)	Pesticides	Occupational
	9			2.12 (1.08–4.16)	Agricultural/horticultural/forestry management	
	2			7.17 (1.78–28.92)	Forestry managers	
	1			7.18 (1.01–51.30)	Horticultural managers	
Kristensen et al. [47]/Norway/Cohort/1952–1991	323,282		Children of farmholders age 0–19 years			One or both parents in agricultural work
	No. cases					
				RR**	**Farming**	
	61	CBT*		1.59 (1.16–2.17)	Pig	
	15	Leukemia other than ALL* or AML*		2.10 (1.07–4.12)		
	30	Neuroepithelial tumors		3.11 (1.89–5.13)	Pig	
	22			2.42 (1.44–4.08)	Chicken	
	25			1.72 (1.05–2.84)	Grain	
	17			2.93 (1.54–5.60)	**Norwegian Krone (NOK) spent on pesticide purchases** 100–499	
	7			3.28 (1.39–7.76)	≥ 500	
Van Maele‐Fabry et al. [45]/Case–control & Cohort Meta‐analysis/1974–2010	N/A	CBT*	Parent	Case–control	Pesticides	One or both parental occupational exposure (farm/agricultural)
				1.30 (1.11–1.53)		
				Cohort (RR**)		
				1.53 (1.20–1.95)		
Kunkle et al. [46]/Meta‐analysis/1966–2010	N/A	CBT*	Parental		Pesticides	Farm related exposure
			Father	2.29 (1.39–3.78)		Preconception
				1.63 (1.16–2.31)		During pregnancy
			Mother	1.48 (1.18–1.84)	Agricultural	
				1.36 (1.10–1.68)	Nonagricultural	
			Child	1.35 (1.08–1.70)	Agricultural	< 19 years of age
				1.32 (1.39–3.78)	Nonagricultural	
Van Wijngaarden et al. [47]/Case–control/USA & Canada/1986–1989	Cs/Ctrl	CBT*	Parent			
	322/321	Astrocytoma				
	51/37		Father	1.6 (1.0–2.7)	Herbicides	During work
	99/85			1.6 (1.0–2.6)	Fungicides	
	53/35		Mother	1.9 (1.1–3.3)	Insecticides	During work
Al‐Buraiki et al. [48]/Case–control/Egypt/2019	Cs/Ctrl 170/170	ALL*	Child ≤ 18 years of age	2.14 (1.04–4.40)	Father works as a farmer	
				5.37 (2.874–10.03)	Agricultural pesticides	
				2.01 (1.128–3.579)	Household pesticides	
Gunier et al. [49]/Case–control/California, USA/1995–2008	Cs/Ctrl	ALL*	Parent		Perinatal	
	699/1021					
	456/548	Child diagnosed < 5 years of age	Father	2.3 (1.3–4.1)		Paternal occupation
	567/885	Child diagnosed < 15 years of age		1.7 (1.1–2.8)		Farm/ranch worker
Vinson et al. [50]/Case–control & Cohort Meta‐analysis	N/A	Leukemia	Mother	1.48 (1.26–1.75)	Occupational (farmers or chemical industry), home and garden use, proximity of residence to agricultural area	Prenatal
			Father	1.32 (1.20–1.46)		
			Both parents	1.84 (1.29–2.44)		
			Mother	2.12 (1.17–3.84)		Postnatal
			Father	1.33 (1.07–1.66)		
			Child	1.85 (1.15–2.96)		“Ever”
		CBT*	Father	1.49 (1.23–1.79)		Prenatal
			Both parents	1.37 (1.08–1.76)		
			Father	1.66 (1.11–2.49)		Postnatal
			Child	1.16 (1.01–1.32)		
			Father	1.41 (1.11–1.79)		“Ever”
		Leukemia	Father	1.37 (1.23–1.52)	Occupational	
		CBT*		1.40 (1.20–1.62)		
		Leukemia	Father	1.26 (1.06–1.49)	Home or garden use	
			Mother	1.56 (1.21–2.02)	Pesticides	
		CBT*	Father	1.48 (1.22–1.80)		
		Leukemia		1.26 (1.14–1.39)	Pesticide class Herbicides	
				1.17 (1.03–1.33)	Insecticides	
		CBT*		1.31 (1.08–1.60)	Herbicides	
				1.18 (1.06–1.33)	Insecticides	
				1.32 (1.06–1.65)	Fungicides	
Efird et al. [51]/Case–control/USA, Israel, Italy, Spain, Australia, France & Canada/1976–1994	Cs/Ctrl 1218/2223	CBT*	Child (< 20 years of age)			Childhood farm/animal exposure
	92/164			1.6 (1.1–2.2)	On a farm < 6 months of age	
					On‐farm animal exposure	
				1.7 (1.0–3.0)	Pigs	
				1.6 (1.0–2.4)	Horses	
				1.5 (1.1–2.0)	Dogs	
				1.5 (1.0–2.1)	Cats	
	75/119		Mother	1.4 (1.0–1.9)	Any farm animal	
				2.3 (1.1–4.9)	Pigs	During pregnancy
				1.8 (1.0–3.1)	Horses	
				1.5 (1.0–2.2)	Poultry	
				1.5 (1.0–2.1)	Dogs	
				1.7 (1.1–2.6)	Cats	
		PNET*	Child	1.9 (1.1–3.2)	Dogs	Childhood farm/animal exposure
	23/164			2.2 (1.2–3.8)	Cats	
		CBT*	Mother	3.8 (1.3–11)	General farm worker	Maternal employment
				2.3 (1.2–4.7)	Employed on farm or in agricultural work involving contact with animals	
				1.9 (1.3–2.8)		
				1.8 (1.1–3.0)	Agricultural chemicals	Job‐related exposure
				2.0 (1.2–3.2)	Fertilizers	
				1.4 (1.1–1.9)	Pesticides	
				2.0 (1.3–3.2)	Animal products	
					Animal manure	

* CBT, Childhood Brain tumors; ALL, Acute Lymphoblastic Leukemia; AML, Acute Myeloid Leukemia; PNET, Primitive Neuro‐Ectodermal tumor.

** RR‐Rate ratio.

Parental agricultural occupation or exposure to fungicides or herbicides was not associated with an increased risk of pediatric brain tumors in a study involving 312 case–control pairs [47]. In this study, a 1.5‐fold increased risk of astrocytoma was observed in children whose mothers were exposed to insecticides [47]. A 2014 meta‐analysis of 15 studies assessing pesticide exposure in utero reported elevated CBT risk for children whose mothers had farm related exposures during pregnancy when compared to maternal exposure to pesticides classified as non‐agricultural [46]. There was also an increased risk of over two‐fold when fathers were exposed to pesticides during pregnancy. These findings are consistent with a previous meta‐analysis performed in 2013 [45]. In this study, when either parent had an expected exposure to pesticides in the workplace (mostly agricultural settings), their children were at increased risk of developing a brain tumor. The result was consistent for case–control and cohort studies included in the meta‐analysis [45].

A Norwegian study reported that children aged zero to 14 years exposed to pesticides and poultry farming had a two‐fold risk for brain tumors and a three‐fold risk for neuroepithelial tumors [52]. Children whose parents worked on farms were also at increased risk. For children of fathers employed in swine production during the conception period, the risk of brain tumors increased three‐fold [52]. A 2001 cohort study conducted in Sweden found a relationship between paternal exposure to pesticides and increased central nervous system tumors in their offspring [44]. The study followed children from birth to 14 years of age and linked the children's cancer diagnosis with paternal occupation obtained through census data. Occupational exposure to pesticides increased risk by 2.5 times but there was no association between childhood leukemia and paternal exposure in this study [44].

Gunier et al. [49] found an association between fathers reporting exposure to pesticides in the workplace during the perinatal period and risk for ALL in children under five. No association was observed for pre‐ or post‐natal maternal occupational pesticide exposure and ALL risk. Children whose fathers worked as farmers or ranchers also had a slightly increased risk for ALL [49]. Subsequently, an increased risk of childhood leukemia was also linked to paternal occupation in an agriculture‐related field [48].

Children whose fathers were certified pesticide applicators and reported not wearing chemically resistant gloves in their agricultural jobs were at almost twice the risk compared to children of fathers who reported wearing gloves [53]. This assessment of pesticide applicators participating in the Agricultural Health Study did not find an increased risk associated with specific types of childhood cancer or with the mixing and application of pesticides. Only paternal use of aldrin showed a statistically significant association with childhood cancer [53].

A meta‐analysis of 38 case–control and two cohort studies reporting OR values and three cohort studies reporting RR values reported no risk of childhood cancer related to parental exposure to pesticides for the three cohort studies reporting RRs [50]. For the other 40 studies, leukemia (acute, lymphoid, or myeloid) incidence was significantly increased for children whose mothers had prenatal exposure to pesticides and to a lesser degree for paternal exposures. The risk of brain cancer was associated with paternal exposure either before or after birth. Interestingly, if both parents had exposure during the prenatal period, leukemia was further increased. The risk of leukemia was higher when the mother was exposed postnatally and when exposed to pesticides in the home or garden. The type of pesticide influences cancer risk. The risk of leukemia was higher for herbicides and insecticides, but not for fungicides. All three classes of pesticides have been linked to brain cancer. The authors did not find an association between increased risk of pediatric leukemia, lymphoma, or brain cancer and living in agricultural areas. However, the father's occupation involving agricultural pesticides increased prenatal brain cancer risk. The mechanism might have involved paternal gene mutations leading to a heritable trait in offspring or epigenetic changes that altered gene function. Alternatively, children might have been exposed through the placenta during development. Overall, exposure to both occupational and household pesticides is significantly associated with increased risks of leukemia and brain cancer in children, and risk for specific cancer types appears to be related to parental exposure during the prenatal period [50].

### 2.6 Genetic Susceptibility

Metabolic enzymes, such as *Cytochrome P450 2E1* (a phase I enzyme) and *NAD(P)H quinone oxidoreductase 1* (a phase II enzyme), play critical roles in xenobiotic metabolism [54]. However, our literature review identified few studies examining their association with pediatric leukemia or brain tumor risk. Toxic intermediates and end products, including those that react to form more harmful compounds, pose increased risks to individuals with specific genotypes that hinder the expression of enzymes necessary for effective metabolism and excretion of these substances. Given the short latency period inherent in pediatric cancer, carcinogenesis may be uniquely linked to high‐intensity environmental exposure and/or highly susceptible genotypes [9].

Some investigators hypothesize that insecticides specifically targeting the nervous system are linked to the development of CBT (Table 5). Cases participating in a case–control study evaluating the risk of pesticides used around and in the home had an increased risk of ALL when their mothers were carriers of *CYP1A1m1* and *CYP1A1m2* mutations, and the mother was exposed to pesticides during her pregnancy [19]. The risk was also increased if the children of mothers with this genetic polymorphism were exposed to indoor insecticides. No effects were observed for the interaction of exposure and *CYP1A1m4*, CYP2D6*3, CYP2D6*4, *GSTM1*, or *GSTT1* gene polymorphisms [19]. *CYP1A1* activates some carcinogens, such as through the glutathione S‐transferase (GST) enzyme pathway, which can be inactivated during phase II metabolism. A subset of GST enzymes (the mu class; GSTM) detoxifies by conjugating harmful substances with glutathione. Genes encoding this enzyme class are polymorphic and can impact susceptibility to chemical toxicity in individuals. Null mutations have been associated with an increased risk of cancer.

**TABLE 5:** Studies on gene–environment interactions due to residential and agricultural pesticide exposure and their associated risk of pediatric brain tumors or leukemia.

Author, year/geographic location/study design/diagnosis year(s)	Number of study subjects	Cancer Type	Exposed person/period of exposure	Odds Ratio (95% CI)	Gene–environment interaction	Genetic factors
Nielsen et al. [55]/Case–control/Washington, USA/1984–1991	Cs/Ctrl 66/236	CBT*	Child (< 20 years of age)			Paraoxonase I (PON1)
	9/10			6.6 (1.5–29.7)	Home treatment for insect pests	PON1_C‐108CT_
						TT
	8/26			2.6 (1.2–5.5)		CT
Infante‐Rivard et al. [19]/Case–control/Quebec, Canada/1980–1993	Case only 123	ALL*	Mother 1 month before pregnancy to end of pregnancy		Pesticides used in the home, garden, yard and/or interior plants	
				5.02 (1.00–25.09)	Mites and spiders	CYPA1m1
				5.55 (1.36/22.67)	Repellants/sprays for outdoor insects	
			Child (< 9 years of age)	3.71 (1.11–12.40)	Rats and mice	
				3.64 (1.19–11.17)	Repellants/sprays for outdoor insects	
			Mother 1 month before pregnancy to end of pregnancy	4.73 (1.18–18.58)	Cockroaches, ants, flies, bees, wasps	CYPA1m2
Chokkalingam et al. [56]/Case–control/California, USA/1995–2002	Cs/ctrl 377/448	ALL*	Mother	3.03 (1.59–5.78)	Indoor insecticide use (prebirth)	
	46/51					ABCB1 G‐A‐G‐T
Medina‐Sanson et al. [54]/Case–control/Mexico//2014–2016	Cs/ctrl	ALL*	Child < 17 years of age		Insecticide exposure of mother after birth (IEAB)	NAT2_rs1041983
	478/284					NAT2_rs1799929
	NR			5.66 (3.92–8.17)		NAT2_rs1799931
						NQ01_rrs1800566
	NR			9.22 (6.24–13.61)	IEAB and smoking by father after birth	

*Note:* NR, not reported.

* CBT‐Childhood Brain tumors; ALL‐ Acute Lymphoblastic Leukemia.

Arylamine N‐acetyltransferase 2 (*NAT2*), cytochrome P450 (*CYP450*) Family 2, Subfamily E Member 1 (*CYP2E1*), and *NAD(P)H quinone oxidoreductase 1 (NQO1)* are xenobiotic metabolizing enzymes that aid the body in detoxifying and eliminating environmental contaminants such as pesticides and *N*‐nitroso compounds. The genetic polymorphisms of these enzymes were assessed for their associated gene–gene and gene–environment interaction on ALL risk in Mexican children [54]. Statistically significant differences were observed in the distribution of *NAT2* gene alleles, and three *NAT2* single‐nucleotide polymorphisms (SNPs) were associated with ALL under recessive and heterozygous models. *CYP2E1* and *NQO1 SNPs* were not associated with ALL. A three‐SNP interaction increased the risk of developing ALL by 6.5 times (CI 4.05–10.7). One *NAT2* genotype was associated with a high risk of developing ALL with exposure to insecticides [54]. Classical genetic traits are inherited, as alleles from both parents combine to form a genotype. These combinations can have protective or harmful effects on cancer outcomes [57]. In cancers resulting from suspected environmental exposures, the focus is on genes involved in xenobiotic metabolism, the body's process of mitigating these exposures, and on epigenetic events. While family history is not strongly associated with pediatric cancer [14], combining alleles in just the right way, passing down damaged DNA, and the time and type of exposure can lead to the development of cancer.

Analysis of genes potentially involved in ALL identified “risk haplotypes”, including three *CYP450* genes, that were more likely to contribute to cancer development when interacting with household chemicals, such as insecticides [56]. *CYP450* genes are associated with the liver's metabolism of pharmaceuticals and other compounds [56]. The significance of these genes in metabolism and xenobiotic processing suggests a potential decisive role in processing various contaminants, and thus, a possible role in mitigating cancer risk. The *CYP2D6* protein produced via transcription and translation exhibits reduced or no activity compared to the wild‐type genotype, rendering it a poor metabolizer, which allows compounds to accumulate. Part of the *CYP1A1* gene was found to have a non‐coding region that increased catalytic activity and the amount of DNA adducts in cord blood and placenta, which increases cancer risk if the adduct is not removed [58].

Among 113 pediatric leukemia patients in Brazil, mutant *CYP1A1* and *CYP2E1* alleles increased the risk of ALL when both were present [59]. Another Brazilian study found that *CYP2D6* was associated with an increased risk of ALL, while genotypes *EPHX1*, *NQO1*, and *MPO* appeared to protect against leukemogenesis [60]. This provides evidence that genetic variability provides protection or harm while raising the possibility of an equilibrium between activation and detoxification that prevents cancer [60]. Their combined effect suggests that xenobiotic‐metabolizing genes influence the development of childhood leukemia.

A study by Sinnet [61] identified metabolic genes that interact with the environment, thereby increasing the risk of cancer. In oxidation activation, the genes for *CYP1A1* and *CYP2D*6 were studied, and the conjugation and detoxification enzymes *GSTM1*, *GSTT1*, *NAT1*, and *NAT2* were linked to environmental interactions. Others report that some genes put children at risk when interacting with their environment, but no conclusive evidence exists to connect gene–environment interaction [7]. *CYP2E1*, *GSTM1*, *NQO1*, *NAT2*, *MDR1*, and *XRCC1* contain genetic polymorphisms that may contribute to leukemia associated with environmental factors [7].

Protein paraoxonase 1 (PON1) polymorphism is linked with susceptibility to brain tumor development [55]. Children with higher PON1 levels can break down toxins from their environments more efficiently than those with lower PON1 levels. This study identified stronger associations in children living in homes with insecticide exposures, exemplifying the gene–environment relationship.

Gene deregulation by translocation occurs when a chromosome segment is relocated to a different chromosome, potentially disrupting regular gene expression. This can activate oncogenes by placing them under the control of highly active promoters or enhancers from other genes, or it can inactivate tumor suppressor genes by disrupting their coding sequences [62]. Studies have identified mutagens capable of changing the developing embryo's DNA through chromosomal translocations [63]. With little known about the latency period for pediatric cancer, Greaves surmised that chromosome translocations arise in utero during fetal hematopoiesis and concluded that most cases of childhood ALL and AML are of prenatal origin [63]. It is unclear whether the translocation is enough to cause cancer or if there is a mediating factor, such as an exposure, that combines with the translocation to drive carcinogenesis. The t(14;18) translocation is common in follicular lymphoma patients [64], but its presentation is unclear, as some healthy individuals also carry this translocation [65]. It is plausible that exposure leads to the translocation, leaving the subject vulnerable to tumor formation due to dysregulation of apoptosis resulting from differences in *BCL‐2* gene expression. Farmers who applied pesticides were at increased risk for t(14;18)‐positive non‐Hodgkin lymphoma (NHL), as were farmers who reported using insecticides and herbicides [65]. NHL risk increased with use; no association was observed between exposure and t(14;18)‐negative NHL [66].

Epigenetics is the study of gene modification that can alter how the cell's machinery reads the gene when transcribing and translating the genetic code to produce proteins. There is evidence that the endocrine‐disrupting pesticides methoxychlor and vinclozolin have epigenetic effects on the germ line, suggesting potential impacts on the reproductive toxicology of this class of compounds. A study done by Manikkam related this to exposures to a gestating mother and the effect it could have on offspring for generations [67]. Tying this to studies showing an increased cancer risk associated with paternal occupation provides a possible epigenetic linkage. If pesticides epigenetically affect parents, modified genes are passed on to their offspring. The researchers were not looking for carcinogenesis, but the ability of pesticides to cause methylation is cause for concern [67]. One study found that epigenetic biomarkers indicating prenatal tobacco smoke exposure were significantly associated with an increased number of somatic gene deletions in childhood B‐cell ALL. Specifically, decreased DNA methylation at the *AHRR* gene and elevated poly‐epigenetic smoking scores were associated with increased gene deletions, indicating a potential mutagenic effect of in utero tobacco exposure in the development of leukemia [68].

While rural migration plays a role in the genetic makeup of a community, people remaining in rural areas build families there, and many stay for several generations. Across generations, exposure to pesticides can lead to alterations in DNA. This genetic damage, including genetic modifications acquired over a lifetime, is passed down [69]. As a family progresses through subsequent generations, the cumulative damage from several generations becomes apparent. This accumulation sets the stage for the potential activation of an oncogene or the initiation of carcinogenesis in a child's metabolism due to exposure to specific pesticides or their combinations [70].

## 3 Discussion

### 3.1 Main Findings

This scoping review demonstrates significant associations between environmental pesticide exposures and increased risks of pediatric leukemia and brain tumors. Residential exposures, most definitively exposures from household insecticide use, are associated with increased risks for both leukemia and brain tumors. Vulnerability to these exposures appeared most pronounced when the exposures occurred during critical windows such as pre‐pregnancy and early childhood. The largest number of studies assessing pesticide exposures associated with risk for childhood leukemia and/or pediatric brain cancer evaluated proximity to agrarian areas (Table 3) and parental workplace pesticide exposures (Table 4). Investigators hypothesized that proximity to farm fields served as a surrogate for pesticide drift to the child's residence or contamination of their water supplies. Specific crops, such as soybeans and oats, were associated with higher cancer incidence rates, suggesting that the types or amounts of pesticides used in their cultivation may play a role. Parental occupational exposure, especially in agricultural settings, also contributed to these risks, with paternal exposure often showing stronger associations (Table 4).

Literature examined here provides evidence that genetic factors contribute to pediatric leukemia risks. Maternal exposure to pesticides during pregnancy carried greater risk of ALL for the child if the mother carried specific polymorphisms affecting metabolism [19, 21]. Several studies examining genotypes of children themselves, which were linked to greater risks of leukemia [56, 58, 59, 60, 61] and brain tumors [55], uncovered evidence that mutations at these loci affect detoxification of contaminant compounds.

### 3.2 Limitations, Gaps, and Recommendations for Future Studies

Some early investigations were hampered by household interviews that captured only pesticide types (insecticides, herbicides, etc.), without rigorously identifying the active ingredients or commercial products used on site [21, 61]. Misclassification of pesticide chemicals at the household level results in weaker or less accurate risk associations. Similarly, risk associations can also be weakened or distorted when cancer cases are classified inexactly. Brain tumors were grouped with other nervous system tumors in many of the studies [43, 45, 46, 50, 51, 52, 55]. In a related limitation of environmental epidemiology, sensitivity to detect associations is constrained by the latency period between exposure and diagnosis. Even for pediatric populations, cancer latency often spans years, heightening the effects of recall bias and misclassification of exposure.

Drinking water is an obvious direct route of exposure to environmental contaminants that could elevate risk of illnesses in local communities. However, we found that few published studies have rigorously examined such associations between pesticides detected in drinking water and risk of childhood leukemia or brain tumors. The investigative limitations underlying this gap in the literature illustrate the complexity of evaluating any of the various routes of exposure (e.g., air, dust, food, bathing, occupational contact, and nonspecified routes accounted for by physical proximity). Methodological limitations include as follows: (1) no mandated monitoring under the Safe Drinking Water Act for many individual pesticides and their transformation products, for which routine voluntary analysis can be prohibitively expensive; (2) difficulty accessing residential history for users of public water systems; (3) lack of requirement for monitoring private well water quality; (4) inconsistent reporting of household water filtration systems and use of bottled water; (5) long‐term cumulative effects of agricultural chemicals present in drinking water at concentrations below reporting thresholds; (6) flawed estimation of drinking water quality by extrapolation from adjacent sources like surface water or irrigation wells. Future studies may be able to establish more reliable conclusions if databases are established to compile agrichemical measurements from water supplies in agricultural‐intensive states, analytical instrumentation is installed at the household level, age‐dating techniques are employed to establish length of exposure, and household surveys are improved to capture more household‐level variables like use of filtration systems and bottled water.

Another clear knowledge gap emerged from this scoping review around the interactions between genetics and specific types of pesticides. Small sample size and recall bias are two limitations commonly mentioned in this subset of studies. This indicates a need to search for new ways to analyze the data and identify genetic factors, particularly for pediatric cancers, which exhibit more genetic influence than adult cancers because of the shorter latency period [21]. Researchers have access to new statistical methods to analyze genetic factors that account for shorter latency pediatric cancers and identify mixtures with the highest impact on the outcome, such as Weighted Quantile Sum, Bayesian Kernel, and Quantile G‐computation. Despite these improvements in statistical methods for evaluating mixtures, challenges persist in determining which risk factors and outcomes are the most critical [71, 72].

Considering the limitations posed by studies that have grouped brain tumors with other nervous system tumors, future studies should report specific types of brain tumors, allowing them to be delineated from other tumors of the central or peripheral nervous system, such as neuroblastoma. Furthermore, future studies should also catalog specific genotypes to enable evaluation of genetic impacts on risk with pesticide exposure. We recognize there may be feasibility concerns with more focused studies due to the large sample size required to achieve adequate statistical power in rare events such as pediatric cancers.

The reports included in this review acknowledge the potential for bias that skews the resulting analyses. Estimating exposure based on self‐report introduces recall bias and can lead to misclassification of exposure. Differences in sampling design, geographical location, exposure assessment, and chemical compounds could partially explain the spurious results among the studies included in our review. Large longitudinal studies with precise repeated measurements of exposure and biomarkers will ultimately be critical for prevention, diagnosis, and treatment of these cancers. Such cohort studies could include quantification of agrichemicals and their combinations in drinking water, while enrolling both children and parents from around the time of birth to track how genotypes shape the mutagenicity of chemical exposures.

Deeper mechanistic understanding is a prerequisite for developing more effective surveillance, risk assessment, and preventive interventions for households and communities, and to mitigate genetic damage in children exposed to agrichemicals. Laboratory research is needed to uncover mechanisms by which genotypes of parents and offspring interact with environmental exposures to change the cancer risk profile. Metabolic and physiological studies are needed to show how pesticides and other chemicals mutate DNA, both in parents and children.

## 4 Conclusion

Even considering the important gaps that remain, this scoping review affirms that a robust body of epidemiology literature already informs how parental and childhood exposure to environmental chemical exposures can be associated with children's incidence of pediatric leukemia and brain cancer. Evidence implying that common practices and circumstances (e.g., household pesticide use and living near intensive agriculture) can increase the rate of potentially lethal pediatric cancers is a call for action and investments in prevention. These might include more robust and targeted surveillance and risk assessment practices, preventive education criteria for households and communities, genetic susceptibility screening, and innovative mitigation practices. Finally, transparency and collaboration among community stakeholders, state and federal health agencies, and academic researchers are essential for robust further research that resolves risk factors and biological mechanisms more clearly and illuminates more powerful intervention strategies.

## Author Contributions

**Grace N. VanDeSteeg:** conceptualization, investigation, methodology, project administration, visualization, writing – original draft, writing – review and editing. **Alyssa R. Russum:** conceptualization, funding acquisition, investigation, writing – original draft, methodology. **Matthew R. Sandbulte:** conceptualization, methodology, visualization, writing – original draft, writing – review and editing. **Eleanor G. Rogan:** conceptualization, investigation, writing – review and editing. **Martha G. Rhoades:** conceptualization, investigation, funding acquisition, methodology, project administration, supervision, writing – original draft, writing – review and editing.

## Funding

This work was supported by the University of Nebraska‐Lincoln.

## Conflicts of Interest

The authors declare no conflicts of interest.
